# A transformation clustering algorithm and its application in polyribosomes structural profiling

**DOI:** 10.1093/nar/gkac547

**Published:** 2022-07-11

**Authors:** Wenhong Jiang, Jonathan Wagner, Wenjing Du, Juergen Plitzko, Wolfgang Baumeister, Florian Beck, Qiang Guo

**Affiliations:** State Key Laboratory of Protein and Plant Gene Research, Peking-Tsinghua Center for Life Sciences, Academy for Advanced Interdisciplinary Studies, School of Life Sciences, Peking University, Beijing 100871, China; Department of Structural Molecular Biology, Max Planck Institute of Biochemistry, Am Klopferspitz 18, 82152 Martinsried, Germany; Department of Cellular Biochemistry, Max Planck Institute of Biochemistry, Am Klopferspitz 18, 82152 Martinsried, Germany; State Key Laboratory of Protein and Plant Gene Research, Peking-Tsinghua Center for Life Sciences, Academy for Advanced Interdisciplinary Studies, School of Life Sciences, Peking University, Beijing 100871, China; CryoEM Technology, Max Planck Institute of Biochemistry, Am Klopferspitz 18, 82152 Martinsried, Germany; Department of Structural Molecular Biology, Max Planck Institute of Biochemistry, Am Klopferspitz 18, 82152 Martinsried, Germany; CryoEM Technology, Max Planck Institute of Biochemistry, Am Klopferspitz 18, 82152 Martinsried, Germany; State Key Laboratory of Protein and Plant Gene Research, Peking-Tsinghua Center for Life Sciences, Academy for Advanced Interdisciplinary Studies, School of Life Sciences, Peking University, Beijing 100871, China; Changping Laboratory, Beijing, China

## Abstract

Improvements in cryo-electron tomography sample preparation, electron-microscopy instrumentations, and image processing algorithms have advanced the structural analysis of macromolecules *in situ*. Beyond such analyses of individual macromolecules, the study of their interactions with functionally related neighbors in crowded cellular habitats, i.e. ‘molecular sociology’, is of fundamental importance in biology. Here we present a **NE**ighboring **M**olecule **TO**pology **C**lustering (**NEMO-TOC**) algorithm. We optimized this algorithm for the detection and profiling of polyribosomes, which play both constitutive and regulatory roles in gene expression. Our results suggest a model where polysomes are formed by connecting multiple nonstochastic blocks, in which translation is likely synchronized.

## Graphical Abstract

**Graphical Abstract:**
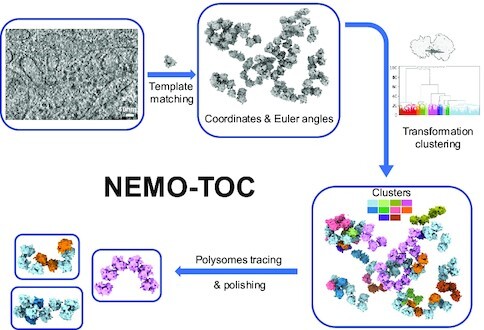
The workflow for NEMO-TOC based polysome tracking and polishing.

## INTRODUCTION

Cryo-electron tomography (cryo-ET) is a recently developed imaging technique allowing three-dimensional representation of the cellular interior, which is preserved in a close-to-native state by vitrification (1). Advances in electron microscopy, sample preparation, as well as computational processing algorithms widen the range of applications of cryo-ET and allow for localization and structural analysis of macromolecular complexes at molecular resolution (2).

Macromolecular interaction networks underly cellular processes. Quantitative investigation of the spatial organization of functionally related macromolecules, i.e. their ‘molecular sociology’, is fundamentally important for studies aimed at understanding their cellular functions (3). With the help of segmentation, subtomogram extraction, and averaging, cryo-ET can provide structural details of individual macromolecules as well as their localizations and interactions (4). Spatial distribution analysis of cryo-ET datasets has been successfully applied to the ubiquitin-proteasome system and the photosynthetic apparatus, providing novel insights into protein homeostasis and photosynthesis, respectively (5–8).

Ribosomes are essential macromolecular machines that translate mRNA-encoded information into proteins. Inside a cell, translational capacity is regulated on the level of individual ribosomes, as well as by other interacting molecules, including neighboring ribosomes. Through interaction with mRNA, ribosomes form higher-order structures named polysomes (9). Irreversible ribosome stalling, caused by damaged mRNA, results in trailing ribosome collisions (10). Under stress conditions, ribosomes may form hibernating dimers as a survival mechanism (11). Because of the polymorphism of polysomes, understanding their higher-order organization remains challenging.

With the advances mentioned above, cryo-ET has the unique potential to address this issue (12–20). Earlier *in vitro* and *in vivo* studies used a manual annotation approach to trace polysomes. However, this workflow is challenging because the mRNA remains invisible. Unlike cases where a spatial distance analysis is sufficient, both distance and angular orientation must be considered in studies of ribosomes. To meet this requirement, an efficient and automated analysis algorithm is needed.

To this end, we developed a NEighboring MOlecule TOpology Clustering (NEMO-TOC) algorithm, allowing the spatial relationship between molecules to be determined by taking into consideration both position and orientation information. Rooted in this concept, we present a polyribosome analysis workflow. Applied to state-of-the-art cellular tomography, we profiled the ribosomal topological organization in primary rat neuron cells. Based on our results, we propose a novel model for polysome formation. Multiple constitutional blocks, within which translation is likely synchronized, are linked together and form one functional polysome. This algorithm could easily be adapted to the structural analysis of other molecular systems.

## MATERIALS AND METHODS

### Grids preparation

Rat cortical neuron grids were obtained by a method described in a previous study (21). Quantifoil grids (R2/1, Au 200-mesh grid, Quantifoil Micro Tools, Germany) were coated with an additional carbon layer (∼20 nm thick) using a carbon evaporator. 250 000 cortical neurons were plated on the grids within 35 mm dishes. The neuron grids were vitrified on 10th day *in vitro* (DIV) with a manual plunger and stored in liquid nitrogen until further processing.

### Cryo-lamella preparation

Grids were mounted onto modified Autogrid sample carriers (22) and then loaded into an Aquilos dual-beam cryo-FIB/SEM (Thermo Fisher) using a cryo-transfer system. A layer of organometallic platinum was applied onto the grid before the further thinning operation to improve the sample conductivity. The lamellas were prepared using a Ga^2+^ ion beam at 30 kV. The beam current was gradually reduced from 0.5 nA to 30 pA during the thinning procedure. SEM imaging was used to monitor the milling progress. The final lamella thickness was between 100 and 200 nm.

### Cryo-electron tomography and reconstruction

The grids with lamellas were examined at a liquid nitrogen temperature using an FEI Titan Krios electron microscope, which was operated at 300 kV and equipped with a Gatan post-column energy filter. Movie frames were collected using a Gatan K2 Summit direct detector operated in dose fractionation mode at a nominal magnification of 42 000×, resulting in a pixel size of 3.42 Å. Tomographic tilt series were recorded using SerialEM software (23) with a cumulative dose of 110 e^–^/Å^2^, covering an angular range between −50° and +70° with an increment of 2°, unidirectionally. Movie frames of each tilt image were motion-corrected using MotionCorr2 (24). Tilt series were aligned using fiducial-free patch tracking, and the tomograms were reconstructed by weighted back-projection using the IMOD software package (25).

### Template matching and subtomogram averaging

The MATLAB (Mathworks)-based TOM toolbox (26) and STOPGAP (27) were used as a general platform for image processing. All tomograms were binned twice, resulting in a voxel size of 13.68 Å^3^ for visualization. Membranes were segmented using a tensor voting-based filter method (28) first, and then manually optimized using Amira (Thermo Fisher). Rat ribosome maps from our previous work were subjected to a low pass filter and severed as templates for ribosome detection using a cross-correlation algorithm in all tomograms with STOPGAP (27). In total, 11 547 ribosomes were selected from the neuron dataset. The resulting coordinates were used to crop subtomograms from full-size tomograms, which were CTF-corrected, aligned, classified, and refined using Warp (29) and RELION v2.1 (30). Different translation states were assigned based on ratcheting status and tRNA occupancy (31,32). The resolution was determined using the 0.143 criteria according to the gold standard Fourier Shell Correlation (33).

### Transformation clustering with NEMO-TOC

**Figure 1.**
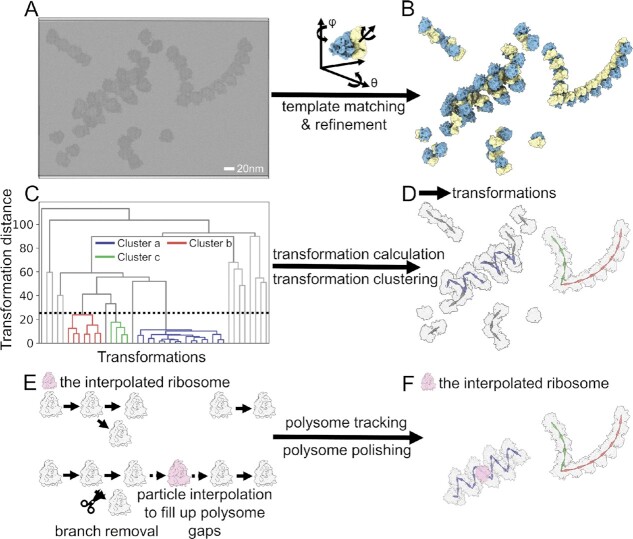
The workflow for NEMO-TOC, polysome tracking, and polishing. The three-dimensional volume of the cellular milieu was reconstructed from cryo-electron tomographic tilt series firstly (**A**). The coordinates and Euler angles of macromolecules of interest were determined by template matching and further refinement (**B**). The transformations between neighboring particles were calculated and used to define a scalar, the transformation distance, to describe the similarity among the transformations. Afterward, hierarchical clustering was adopted to cluster transformations (colored in purple, red and green) out of the non-meaningful cluster background (colored in grey) based on transformation distance (**C**, **D**). Next, polysome tracking was performed by linking connectable transformations within each cluster (**E**). We further refined the polysomes by linking shorter polysomes using an interpolation-based approach and removing polysome branches (E), which resulted in the three polysomes colored in purple, green and red, respectively (**F**).

The inputs for NEMO-TOC are the position and orientation information of each particle (in this study, each ribosome) determined using the methods mentioned in the last section. To better work with the RELION workflow, NEMO-TOC was designed to directly read the star file formatted in RELION. NEMO-TOC extracts the coordinates and orientation information of each particle, after which the original coordinates are updated with the shifts from the refinement to recenter the particles. Firstly, NEMO-TOC searches the spatial neighboring particle pairs using the Euclidean distance threshold given by the user (Supplementary Figure S1A). Secondly, the transformation between two particles in each pair in the search results is calculated with consideration of the position and orientation of each particle of the pair (Supplementary Figure S1B). Transformation distances are further calculated to quantitatively describe the similarity between those transformations (Supplementary Figure S1C). Finally, classic hierarchical clustering is adopted to cluster those transformations using a user-provided threshold cut-off (Figure 1C, Supplementary Figure S1C, D). Thus, particle pairs which share similar transformations are clustered. NEMO-TOC then outputs detailed transformation information for each cluster (translocation vectors and rotation angles) and groups the particles according to the detected clusters into star files. The generated output star files can be readily used for further analysis in RELION or adapted for other subtomogram averaging software.

Considering the high concentration of ribosomes inside the cell, it is possible that some ribosome pairs could show similar transformations coincidentally, so NEMO-TOC further filters non-meaningful clusters by a user-provided threshold. For our study of polysomes, a threshold of 0.5% of the total number of neighboring particle pairs was used based on the simulation experiment mentioned in the next section. This threshold might need to be adapted by the user based on the specific biological problem.

We have uploaded NEMO-TOC to GitHub. A detailed tutorial and a demo dataset are available (https://github.com/GuoQLabPKU/polysomeTracking).

### Simulated dataset analysis

The metric to filter false-positive clusters was investigated with a simulated dataset. In a volume of 1270 nm × 1270 nm × 342 nm, which is the typical size of a tomogram, we randomly positioned 5000 particles using an in-house developed Python script, resulting in a ribosome concentration of 15 μM and around 6000 pairs of transformations. The pairwise transformation distances follow a positively skewed distribution (Supplementary Figure S2A). This dataset was clustered with a linkage threshold of 25 (also used for the *Escherichia coli* dataset), resulting in ∼2000 clusters. The largest cluster contained 12 transformations, which correspond to $<$0.5% of the total transformations (Supplementary Figure S2B). As a result, 0.5% was used as the cutoff to filter false-positive clusters.

### Polysome tracking and polishing

Given the bidirectional nature of each transformation, the transformation direction was aligned before polysome tracking so that only a single consistent direction was kept. Within each cluster, the direction alignment was performed by: (i) randomly selecting one direction for each transformation; (ii) clustering the transformations into two groups in the translocation vector space using the *k*-means method; (iii) selecting the larger group; (iv) reversing the direction of the smaller group (Supplementary Figure S3, step one and two). For translating ribosomes, because of the polarity of the mRNA, in steps (iii) and (iv), only the mRNA 3′to 5′ (mRNA entry site towards the mRNA exit site) direction was reversed.

To track polysomes, after direction alignment, for each transformation *i*, the next connectable transformation *i* + 1 was determined. Transformations *i* and *i* + 1 are considered to be connected if the tail particle of *i* is the same as the tip particle of *i* + 1. This process was iterated until no connected transformations were found (Supplementary Figure S3, Step three).

As transformation clustering of noisy data is never perfect, false-positive detection might lead to branched polysomes, which are caused by transformations that begin or end with the same particle (Supplementary Figure S4A). Thus, we developed a ‘scissor’ to cut branches, which was achieved by keeping only the best scoring transformation for each branching point (Supplementary Figure S4A). After this branch trimming process, another round of polysome tracking was executed without any branching.

An interpolation-based gap filling step was integrated to counteract the possibility of misdetection or misalignment of particles (Supplementary Figure S4B). At the tail of each polysome, a ribosome was interpolated using the average transformation of the cluster that formed the polysome. If the transformation (*trans_int_*) between an interpolated particle and the tip particle of another polysome is comparable with the average transformation within the same cluster, the interpolated particle will be accepted and could be manually verified in the tomogram. To identify if *trans_int_* is comparable with the average transformation, firstly, the distance *dist_int_* of *trans_in**t**_* and the average transformation is calculated. Secondly, the distribution of distances between all transformations and the average transformation is calculated. If *dist_int_* is smaller than the upper 95th percentile (which can also be provided by the user) of this distribution, the interpolated particle is accepted (Supplementary Figure S4B).

### mRNA path deduction

Successful tracking of polysomes allows the deduction of the mRNA path. To achieve this, firstly, the A site and the E site of each ribosome in a polysome were positioned. Then, the mRNA path inside one ribosome was inferred by the direct connection between the E site and the A site, and the mRNA path between neighboring ribosomes was inferred by the direct connection between the E site of the upstream ribosome and the A site of its downstream neighbor. All the positions of mRNA in this work were deduced in this way, and the term mRNA chain was adopted for the sake of simplicity.

### Helix fitting of the mRNA chain

To obtain the helical parameters of the mRNA inside one polysome, we used the program *HELFIT* (34) to fit a standard helix into the mRNA chain and acquired the helical parameters, including the diameter, the pitch, the number of particles per turn, and the fitting root mean square deviation (RMSD).

### mRNA curvature measurement

**Figure 2.**
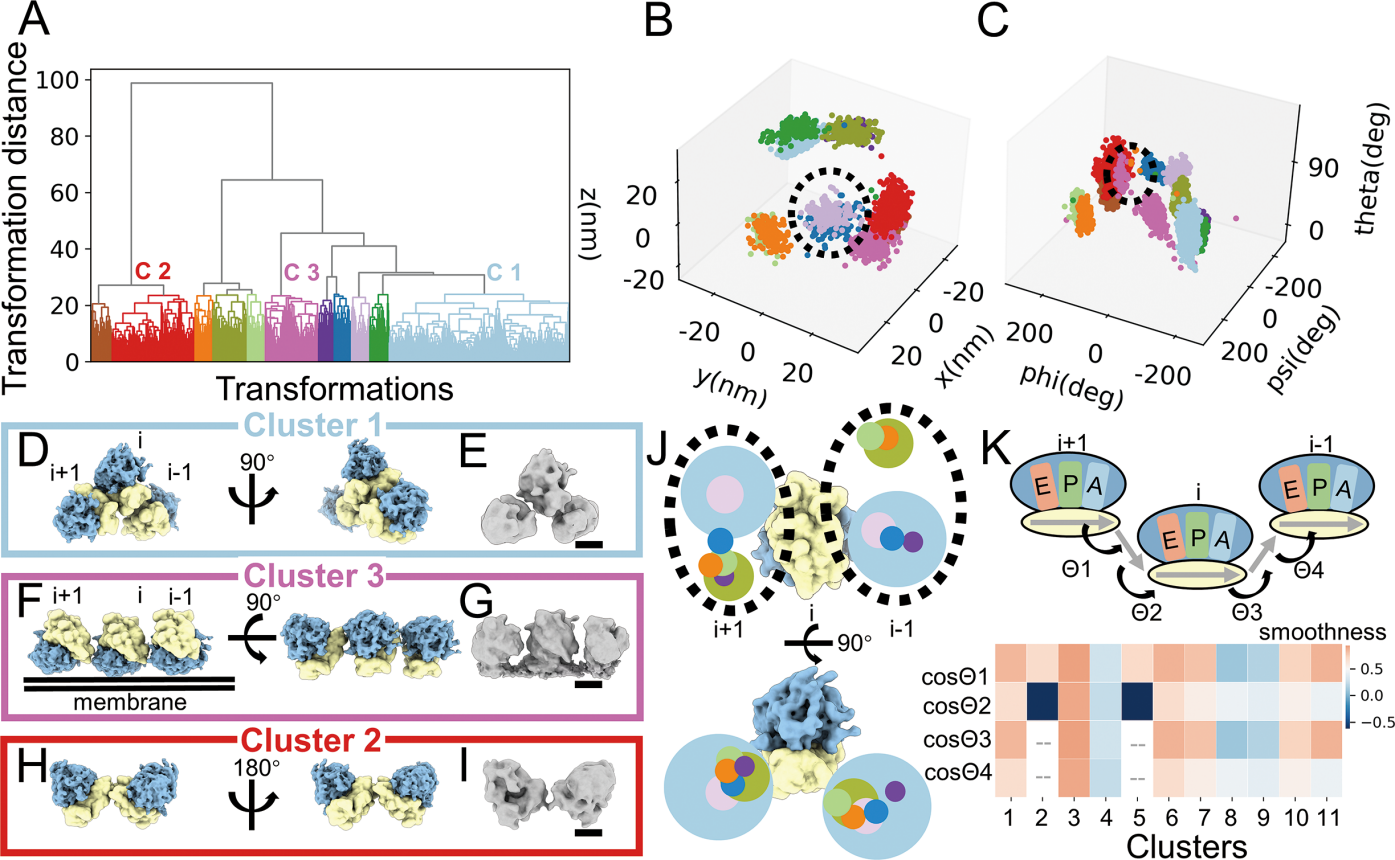
NEMO-TOC of neuronal ribosomes. (**A**) The hierarchical clustering results of the neighboring ribosome transformations. Eleven transformation clusters were clustered from the whole neuronal dataset. Transformation Cluster 1, Cluster 2 and Cluster 3 were the most proportional transformations. (**B**, **C**) The eleven transformation clusters from (A) are not separated perfectly in the translocation vector space (B) or the rotation vector space (C). The dotted circles indicate the overlapped clusters in (B) and (C). (**D, F, H**) The neighboring ribosome arrangements of Clusters 1, 3 and 2 are displayed by the forward models in solid surface representation in two different views. The 40S small subunits of ribosomes are colored in yellow and the 60S large subunits are colored in blue. The upstream and downstream neighboring ribosomes on the mRNA are indicated by *i* + 1 and *i* −1. (**E, G, I**) Direct reconstructions of particles from denoted clusters are displayed in solid surface representation in the same view as (D, F and H). Scale bars: 10 nm. (**J**) The spatial arrangement of all cytosolic transformation clusters except the di-ribosomes (Cluster 2 and Cluster 5). For a given ribosome, which is shown in a solid surface, the arrangements of the upstream (*i* + 1) and downstream (*i* − 1) neighboring ribosomes are represented as circles. The center of each circle represents the center coordinate of the neighboring ribosome while the diameter represents the relative population of the transformation cluster. (**K**) A model of three neighboring ribosomes is shown with A, P and E tRNA sites annotated with different colors. Grey arrows are used to represent the mRNA, and the arrowheads point to the 3′ direction of the mRNA. The curvature of mRNA chains can be quantified by the cosine values of the angles of adjacent mRNA fragments separated by neighboring ribosomes. The angles of adjacent mRNA fragments are labeled as Θ1, Θ2, Θ3 and Θ4. The cosine values of these four angles from different transformation clusters are shown in the heatmap. Boxes with grey dash lines in the heatmap represent missing values for Cluster 2 and Cluster 5, because of their pseudo-C2 symmetry.

The curvature of the detected mRNA chain inside the generated forward models was calculated by the following steps. First, four angles formed by adjacent mRNA fragments inside or between three neighboring ribosomes were detected (Figure 2K). Second, the cosine values of these angles were calculated. Intuitively, a cosine value closer to 1.0 indicates less curvature of the mRNA chain. Therefore, the cosine values of these four angles were used to reflect the curvature of the mRNA. For clusters 2 and 5, because of the di-ribosomes' pseudo C2-symmetry, only two angles were formed by adjacent mRNA fragments for further analysis.

### Figure rendering

All figures were rendered using either Python or ChimeraX (35).

## RESULTS

### Rationale and overall design of NEMO-TOC

The spatial relationship between neighboring molecules can be described by rotation and translocation from one to another. To accurately quantify this information, it is essential to obtain the molecules’ coordinates, as well as their Euler angles against the same reference structure. For cryo-ET samples, these coordinates and angles are usually determined by template matching (36) and subsequent refinement (37).

To describe and classify the spatial relationship, a clustering approach is needed. In three-dimensional space, such an approach involves the clustering of variables of both translational vectors and rotational vectors. However, rotation angles and spatial translocation do not have the same units or ranges, so they are not directly comparable. To overcome this obstacle, Pfeffer et. al defined two comparable angles α and β, which describe the relative positions and rotations of neighboring ribosomes for further clustering (15,17). This method can be implemented only if neighboring ribosomes are in the same plane, as in the case of membrane-bound ribosomes. Another approach (16) defines three comparable distance vectors to include the information of spatial arrangements of neighbors followed by classical K-means clustering; however, this approach requires the number of clusters to be known in advance, and the clustering method itself is sensitive to outliers. These limitations can be overcome by adopting different clustering strategies, such as the hierarchical clustering method used by Brandt *et al.* (38), which does not require prior knowledge of the number of classes. However, a major limitation of this method is the invariance of the metric to the spatial transformation direction, which reduces its detection accuracy. To overcome these drawbacks, we developed NEMO-TOC, a direction-sensitive hierarchical clustering algorithm (Figure 1, Supplementary Figure S1). The method was extended to track ordered topologies and especially linear assemblies with polarity (e.g. polysomes). Furthermore, modules to accomplish the interpolation of undetected particles and automated removal of non-meaningful clusters were added. The workflow was implemented as a python module (https://github.com/GuoQLabPKU/polysomeTracking), which supports GPU acceleration and parallel computation.

### Transformation calculation

The inputs to the NEMO-TOC algorithm are the positions and orientations of individual particles (Figure 1A, B). For each of these datasets, the Euclidian distances to neighbors are calculated. The number of neighbors for the following analysis can be pruned by a distance threshold, which reduces the number of calculations and avoids potential second-order relations, allowing the results to be interpreted more easily. For the ribosome dataset, the distance threshold was set to match the ribosome's diameter (Supplementary Figure S1A).

For each particle *i*, the transformation to its neighbor *k* was calculated including both relative translocation *Vtr* and relative rotation *Mtr*. The relative translocation is defined by rotating the translocation vector (*Pos*_k_ − *Pos_i_*) against the orientation (*M_k_*) of the particle *k* relative to a reference structure (1). For the relative rotation, the rotation difference is calculated (2). To make the clustering algorithm independent of the particle transformation direction (***i→k*** or ***k→i***), it is necessary to calculate the backward transformations as well (3, 4) (Supplementary Figure S1A, B). Thus, for one pair of neighbors, the transformation has two directions.

(1) $$\begin{equation*}Vt{r_{Forward \, i - >k}}\, = \,\left( {Po{s_k} - Po{s_i}} \right) * {M_k}{^{-1}}\end{equation*}$$

(2) $$\begin{equation*}Mt{r_{Forward\, i - >k}} = \ \,{M_i}* {M_k}^{ - 1}\end{equation*}$$

(3) $$\begin{equation*}Vt{r_{Backward\, k - >i}}\, = \,\ \left( {Po{s_i} - Po{s_k}} \right) * {M_i}^{ - 1}\end{equation*}$$

(4) $$\begin{equation*}Mt{r_{Backward\, k - >i}}_{\ \ } = \ {M_k}* {M_i}^{ - 1}\end{equation*}$$

*M* *_i_*: rotation matrix of particle *i* related to the reference

*M* *_k_*: rotation matrix of particle *k* related to the reference

*Pos* *_i_*: coordinate of particle *i* in the tomogram

*Pos* *_k_*: coordinate of particle *k* in the tomogram

### Transformation distance calculation and hierarchical clustering

For clustering, we define a scalar named the transformation distance, which is composed of the translocation distance and the rotation distance. Firstly, distances are calculated separately for rotation and translocation (Supplementary Figure S1C). As a consequence of the bi-directionality of our transformation calculation, transformation distances from all permutations of forward and backward directions must be computed. These distances are combined by a minimum operation using the formulas shown below (5, 6) (Supplementary Figure S1B, C). The individual distance matrices (DistVect, DistRot) are combined by scaling the values of the translocational distance matrix to the values of the rotational distance matrix. Finally, the average of the scaled distance matrix and the rotational distance matrix is computed (7), which generates reproducible values that can be interpreted as angular degrees (pseudo degrees).

(5) $$\begin{eqnarray*} DistVect(m,n) &= &{\rm{min}}({\left| {\left| {{\boldsymbol{Vt}}{{\boldsymbol{r}}_{Forward,m}} - {\boldsymbol{Vt}}{{\boldsymbol{r}}_{Forward,n}}} \right|} \right|_2},\nonumber\\ &&\;\;\;\;\;\;\;\;{\left| {\left| {{\boldsymbol{Vt}}{{\boldsymbol{r}}_{Forward,m}}-{\boldsymbol{Vt}}{{\boldsymbol{r}}_{Backward,n}}} \right|} \right|_2},\nonumber\\ &&\;\;\;\;\;\;\;\;{\left| {\left| {{\boldsymbol{Vt}}{{\boldsymbol{r}}_{Backward,m}} - {\boldsymbol{Vt}}{{\boldsymbol{r}}_{Forward,n}}} \right|} \right|_2},\nonumber\\ &&\;\;\;\;\;\;\;\;{\left| {\left| {{\boldsymbol{Vt}}{{\boldsymbol{r}}_{Backward,m}}-{\boldsymbol{Vt}}{{\boldsymbol{r}}_{Backward,n}}} \right|} \right|_2}) \end{eqnarray*}$$

(6) $$\begin{eqnarray*} && DistRot(m,n) = \nonumber\\ && \;\;\;\;\;{\rm{ min}}\left({{\rm acosd}}\left( {{\rm{trace}}\left( {{\boldsymbol{Mt}}{{\boldsymbol{r}}_{Forward,m}}*{\boldsymbol{Mt}}{{\boldsymbol{r}}_{Forward,n}}{{^ - }^1}} \right){\rm{ }} - {\rm{ }}1} \right){\rm{ }}/{\rm{ }}2,\right.\nonumber\\ && \;\;\;\;\;\;\;\;{\rm{acosd}}\left( {{\rm{trace}}\left( {{\boldsymbol{Mt}}{{\boldsymbol{r}}_{Forward,m}}*{\boldsymbol{Mt}}{{\boldsymbol{r}}_{Backword,n}}^{-1}} \right){\rm{ }} - {\rm{ }}1} \right){\rm{ }}/{\rm{ }}2,\nonumber\\ && \;\;\;\;\;\;\;\;{\rm{acosd}}\left( {{\rm{trace}}\left( {{\boldsymbol{Mt}}{{\boldsymbol{r}}_{Backward,m}}*{\boldsymbol{Mt}}{{\boldsymbol{r}}_{Forward,n}}^{ - 1}} \right){\rm{ }} - {\rm{ }}1} \right){\rm{ }}/{\rm{ }}2,\nonumber\\ && \;\;\;\;\;\;\;\;\left.{\rm{acosd}}\left( {{\rm{trace}}\left( {{\boldsymbol{Mt}}{{\boldsymbol{r}}_{Backward,m}}*{\boldsymbol{Mt}}{{\boldsymbol{r}}_{Backward,n}}{{^ - }^1}} \right){\rm{ }} - {\rm{ }}1} \right){\rm{ }}/{\rm{ }}2\right) \end{eqnarray*}$$

(7) $$\begin{eqnarray*} && Dist(m,n) = (DistRot(m,n)\nonumber\\ && \quad + DistVect(m,n)*{\rm{ }}180{\rm{ }}/{\rm{ }}\left( {2{\rm{ }}*{Radius}} \right)){\rm{ }}/{\rm{ }}2\end{eqnarray*}$$

*DistVect(m,n)*: translocation distance between transformations *m*, *n*

*DistRot(m,n)*: rotation distance between transformations *m*, *n*

*Dist(m,n)*: combined distance between transformation *m*, *n*

*Radius:* search range for neighbors

A hierarchical clustering method was then applied to cluster these transformations based on the calculated distances (Figure 1C, D, Supplementary Figure S1D). For highly populated molecules like ribosomes, a substantial percentage of pairs will be coincidentally grouped to produce non-meaningful clusters (Supplementary Figure S2). To make interpretation of the clustering results more straightforward, these clusters were removed based on the number of transformations they contained. A threshold of 0.5% of the dataset size was determined by clustering simulated data with random orientations. The remaining transformations were subjected to another round of clustering (detailed in Materials and Methods).

### Detection and polishing of ordered linear assemblies

By alignment of transformation direction and subsequent connection of overlapping particle pairs, we can detect ordered assemblies of macromolecules (detailed in Materials and Methods, Figure 1E, F, and Supplementary Figure S3). Here we focus on the performance of this method on linear assemblies with polarity, such as polyribosomes. In cellular cryo-ET, the initial particle positions are subject to misalignment and may not be detected in the first instance due to factors like the low signal-to-noise ratio of the tomograms, missing wedge artifacts, imperfect alignment of tilt series, and molecular crowding, leading to branching and incomplete detection of assemblies. To mitigate these problems, we developed a polishing step with both branch removal and gap filling functions for linear assemblies like polysomes (Figure 1E, Supplementary Figure S4A, B).

### Transformation clustering of neuronal ribosomes

Previous three-dimensional structural analyses of both prokaryotic and eukaryotic polysomes have shown that polysomes likely form relatively stable conformations, represented by helical, circular, and zigzag configurations (12–14,16). Compared to prokaryotes, eukaryotes have a more complex cellular environment with different organelles harboring specific functions. The ribosome carries out translation either freely in the cytoplasm or on the surface of different membranes, including the nuclear envelope (39), endoplasmic reticulum (ER) (15), and mitochondria (17). Uncovering the relationship between the spatial arrangement of neighbors and the cellular environment where they are located could provide insight into subcellular location-specific organization principles.

We collected tomograms in the cell body of rat primary neuron cultures. Using a template matching approach, we detected 11,547 ribosomes in 18 tomograms. Upon averaging and classification, resolutions from 12 to 22 Å were obtained showing five distinct conformations, as judged by ratcheting status and occupancy of tRNA sites (Supplementary Figure S5A, B). After transformation clustering and false-positive filtering, we detected 11 clusters (Figure 2A–C, Supplementary Table S1) comprising 68% of all ribosomes, indicating that the majority of detected ribosomes had an ordered relationship with their neighbors. As expected, the transformation distances from different clusters were much larger than those within the same cluster (Supplementary Figure S6). Transformations within the same cluster were clustered tightly both in rotation angle and translocation vector space (Figure 2B, C). However, detailed inspection showed that the clusters would not be perfectly clustered if the translocation vector or rotation was used alone. For visualization, forward models of each cluster were generated by placing neighboring ribosome maps together according to the averaged rotation and translocation (Figure 2D, F, H, Supplementary Movie S1).

For Cluster 1, which is the largest class and contains about 45% of all clustered ribosomes, the upstream ribosome (*i-1*) and the downstream ribosome (*i + 1*) are located at the mRNA exit and mRNA entry direction of a chosen ribosome (*i*), suggesting that they bind to the same mRNA chain and perform translation simultaneously (Figure 2D). We re-extracted the central ribosomes with a larger box size (180 pixels) of bin 2 (6.84 Å/pixel), which includes the densities of neighboring ribosomes, after which we performed reconstruction directly using the alignment parameters of ribosome *i*. This procedure resulted in a map with identical tri-ribosome densities with the forward model (Figure 2E), confirming the robustness of the NEMO-TOC algorithm.

In comparison with Cluster 1, Cluster 3, which contained 11% of all clustered ribosomes, the 40S small subunits of neighboring ribosomes were positioned side-by-side (Figure 2F, G). This finding is consistent with the previously reported spiral packing (38). We found that the peptide exit channels of neighboring ribosomes were on the same plane, suggesting that neighboring ribosomes are subject to external constraints. The ribosomes in Cluster 6 were also restrained to a plane, with neighboring ribosomes moving 22° inwards towards its small subunit (Supplementary Figure S7A). Direct subtomogram reconstruction of the 1166 particles from these two clusters shows a clear membrane density attached to the 60S large subunit and additional densities attached to the peptide exit channels, reminiscent of the membrane-bound ribosome-translocon complex (15). The translocon-associated protein (TRAP) complex and oligo-saccharyl-transferase (OST) complex are visible in the density map (Supplementary Figure S7B), and the positions of these ribosomes in the original tomograms show clear ER localization. It should be mentioned that we did not obtain the membrane-bound ribosome structure by direct classification of all ribosomes after exhaustive attempts, which was likely due to the fact that only a minor fraction (1166 out of 11 547 particles) of ribosomes are membrane-bound in our dataset. This finding highlights the sensitivity of NEMO-TOC.

Cluster 2 (23% of all clustered ribosomes), for which we could not detect a second neighbor, showed a ribosome dimer with a pseudo-C2 symmetry (di-ribosome) (Figure 2H, I). The forward model of Cluster 5 is also a di-ribosome with a spatial shift compared with Cluster 2 (Supplementary Figure S8A). The di-ribosome interface is mediated by the body of the small subunit (h10, Supplementary Figure S8B), which is different from the collided di-ribosome (Supplementary Figure S8C) (40). The existence of hibernating ribosomes in eukaryotes is controversial (41). The di-ribosomes of Cluster 2 and Cluster 5 show a packing mode different from that of hibernating ribosomes in *E. coli* (Supplementary Figure S8D), and no additional densities corresponding to possible hibernation factors were observed (42). Both direct reconstruction and positions in the tomogram indicate cytosolic localization of the di-ribosomes of Cluster 2 and Cluster 5.

The averages of the ribosomes from the remaining clusters did not show membrane densities, indicating cytosolic localization. A plot of the spatial distributions of all of the cytosolic clusters showed that the two neighboring ribosomes of each center ribosome are flopping around its mRNA entry and exit directions. The positions of the neighboring ribosomes are clustered on several regions almost on the same plane (Figure 2J), suggesting that polysome organization involves inter-ribosomal contacts rather than simple tethering by a single mRNA chain only.

For all clusters, we analyzed the conformations of individual ribosomes (Supplementary Figure S5C). No obvious difference was found across these transformations, which implies that they are all translationally active. However, the deduced mRNA trajectories among different clusters showed dramatic variations (Figure 2K). While Cluster 1 and the two membrane-bound clusters showed a smooth connection, the others showed more curved connections, which may reflect the widespread secondary structures of mRNA (43,44). Notably, the mRNA trajectory smoothness, while defining the overall shape of the polysome, may also have a role in gene expression regulation (45).

These results indicated that neighboring ribosome transformations exhibit distinct organization, which may serve as basic blocks in aspects of both constitution and regulation.

### Polysome tracking in neurons

**Figure 3.**
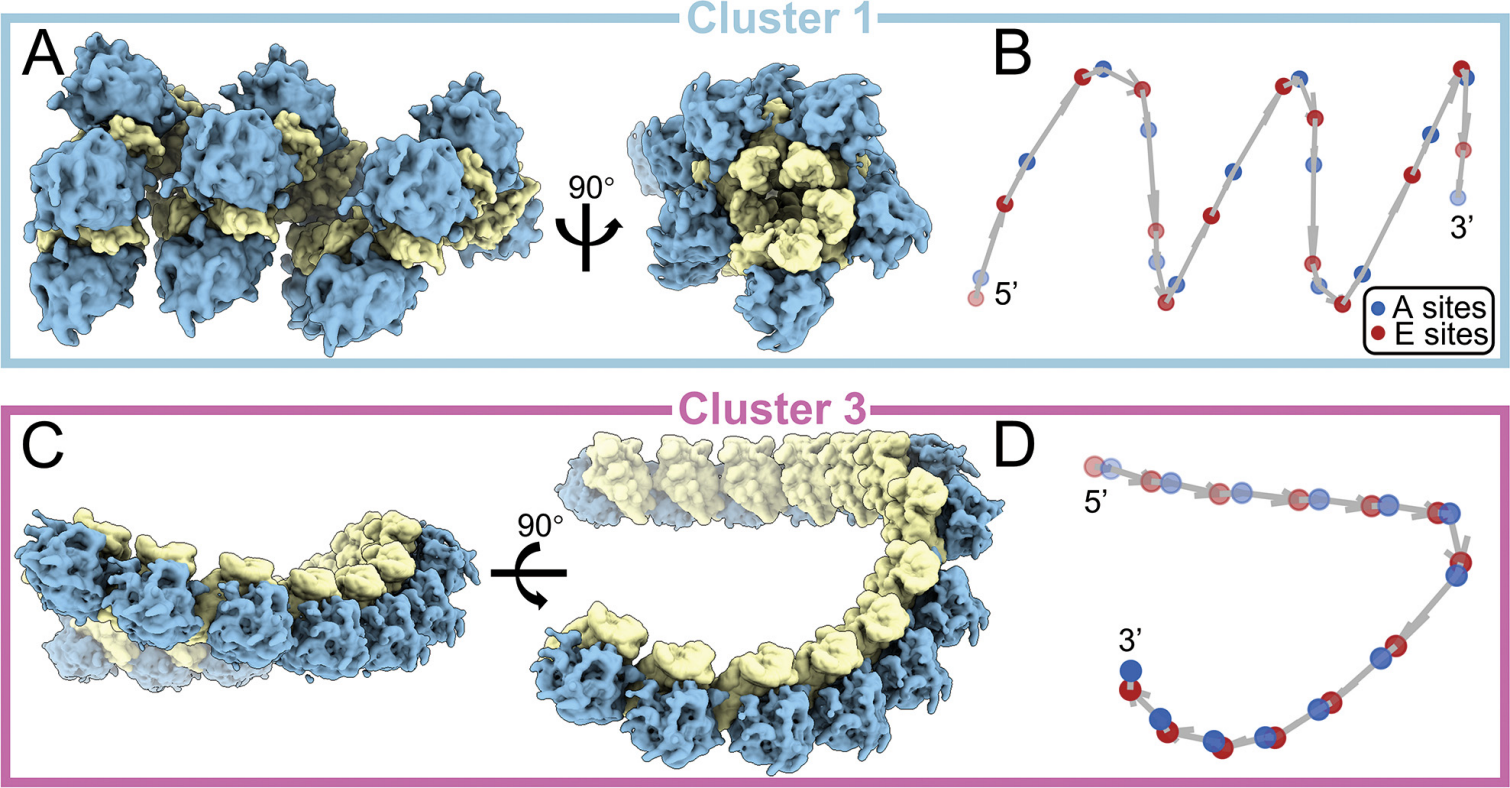
Polysomes formed by an identical transformation cluster. (**A, B**) Transformations from Cluster 1 can be linked into long helical polysomes. (A) shows the longest polysome detected with only Cluster 1 transformations. (B) shows the deduced mRNA chain. The E sites and A sites of ribosomes are colored in red and blue, respectively. (**C, D**) Transformations from Cluster 3 can be linked into long planar polysomes. (C) shows the longest polysome detected with only Cluster 3 transformations. (D) shows the deduced mRNA chain. The E sites and A sites of ribosomes are colored in red and blue, respectively.

The high demand for translation products requires as many ribosomes bound to the same mRNA as possible. As a result, polysomes are believed to be well regulated and form stable higher-order structures, as several studies have reported (12,16,38). Based on the transformation clustering results, we initially tried to track polysomes within each cluster group (Supplementary Figure S3). Cluster 2 and Cluster 5 were excluded because of pseudo C2-symmetry. This procedure resulted in polysomes with a variety of lengths; however, polysomes longer than five ribosomes were detected only in Cluster 1 and Cluster 3 (Figure 3).

In Cluster 1, the longest polysome comprised 14 ribosomes that adopted a left-hand helical conformation with about five ribosomes per turn (Figure 3A). The pitch, the rise, and the diameter of the mRNA trajectories are 345, 71 and 346 Å, respectively (Figure 3B), which are similar to previously reported eukaryotic polysomes *in vitro* except for the smaller diameter (∼570–580 Å) (12). The small subunits of ribosomes are packed inside the helix, and peptide exit channels are exposed to the cytoplasm, which is consistent with previous observations from different species (12,16,38), suggesting an evolutionally conserved polysome topology. With this helical packing, the mRNA is packed inside a large assembly, which protects mRNA from RNase digestion, and may also prevent the formation of complex secondary structures.

For Cluster 3, which represents the case of membrane-bound ribosomes, polysomes with up to 13 ribosomes were detected. Because of the constraints imposed by the ER membrane, the polysome and the mRNA trajectory adopt a planar conformation, with the peptide exit channels facing the ER (Figure 3C, D). This finding is consistent with previous studies of purified ER-derived vesicles (15).

**Figure 4.**
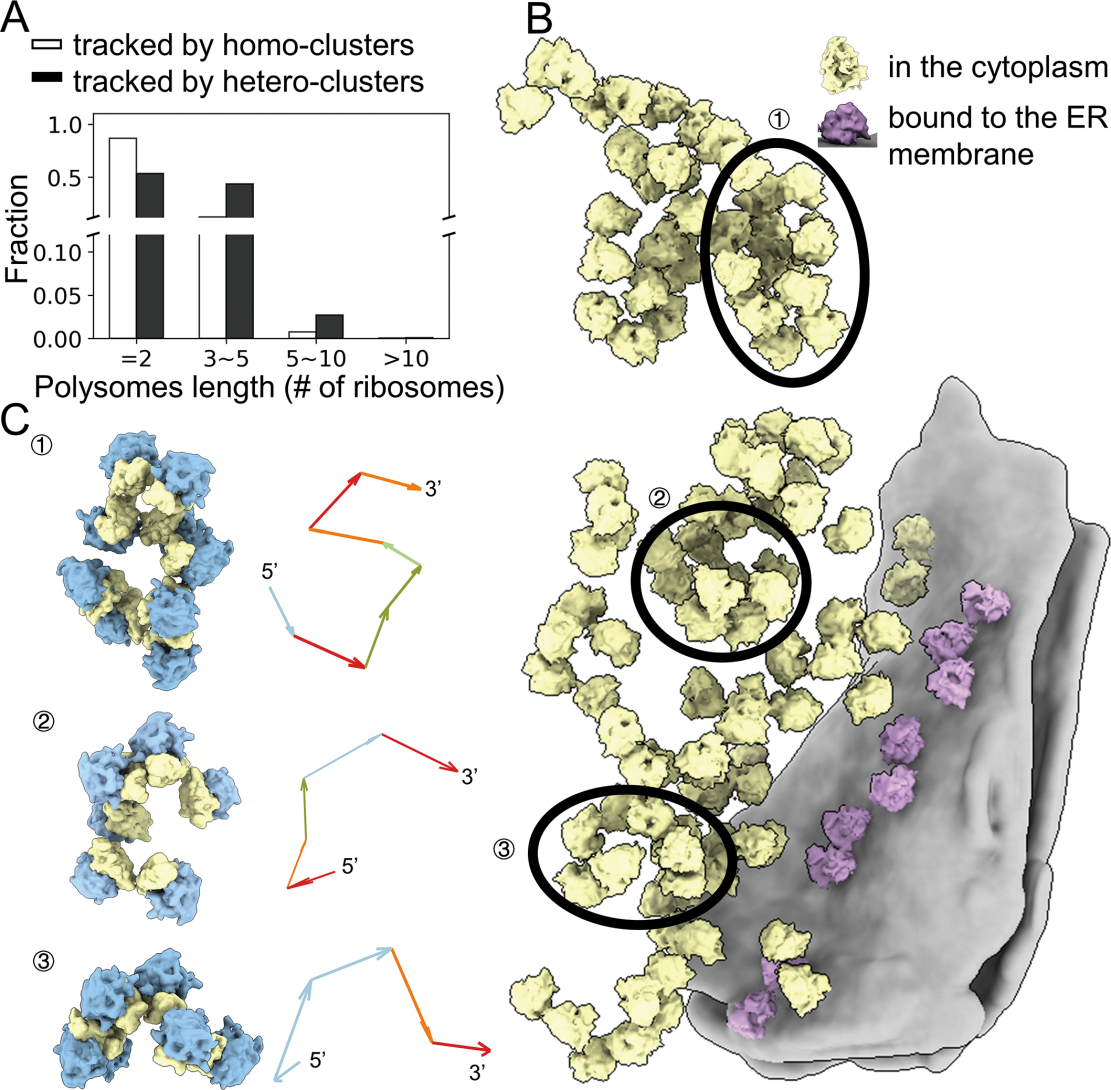
Combinations of different transformation clusters can form long polysomes with high curvature. (**A**) Comparison of polysome length tracked by homo-cluster connections and hetero-cluster connections. (**B**) 3D rendering of ribosomes within a neuron cell. The cytosolic ribosomes are colored in yellow while the membrane-bound ribosomes are colored in pink. Three representative polysomes 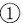, 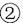 and 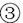 are annotated with ellipses and rendered in (**C**). The mRNA trajectories are annotated with arrows, the color of which is consistent with that of the transformation clusters in Figure 2. The arrows were aligned from the 5′ to 3′ direction of a given mRNA.

It is not surprising that no long polysomes were detected in some of the clusters, as the forward models generated using averaged transformation vectors could not be extended further due to severe steric clashes. Even for the clusters where long polysomes were formed, the majority of polysomes were short (Figure 4A). One hypothesis is that long polysomes were interrupted by undetected ribosomes during template matching, misalignment during subtomogram averaging or removal of material during FIB milling. If this is the case, then those undetected particles should be easily recovered because of their regularity of spacing. This hypothesis was rejected with the following interpolation experiments within Cluster 1. At both the tail and the tip of each polysome with a length of at least three ribosomes, five more ribosomes were interpolated using the average transformation of Cluster 1. All of these interpolated positions were then manually inspected in the original tomograms. Of the 2180 interpolated positions, $<$1% were indeed undetected particles, and the remaining positions were either empty or already annotated with other clusters (Supplementary Figure S9). The interpolation experiment suggests that few particles were missing, and it is unlikely that short polysomes were interrupted by potentially missing particles.

Alternatively, longer polysomes could be built up by hybridized transformations. This hypothesis is supported by the significant proportion of overlapping ribosomes among different clusters (Supplementary Table S2). Indeed, by linking transformations from different clusters, longer polysomes could be tracked (Figure 4A). The combination of different transformation pairs showed no preference, suggesting that polysomes are more likely to be formed by locally ordered blocks of ribosomes rather than as long-range continuously ordered structures. This tendency could lead to the bending of otherwise straight helical polysomes (Figure 4B, C).

## DISCUSSION

In this work, we established a NEMO-TOC algorithm for the topological analysis of macromolecules identified in cellular cryo-ET datasets, which will be of general interest for other *in situ* molecular sociology analyses. We present here a workflow for topological analysis using position and orientation information determined directly from typical subtomogram averaging. Our results show that in the case of ribosomes, the weighted combination of both position and orientation information contributes to efficient topological clustering (Supplementary Figure S10). It has been established that misalignment may occur during subtomogram averaging. Therefore, we tested the sensitivity of the NEMO-TOC algorithm to angular errors by introducing random rotation noise to a simulated dataset. We found that most ribosome pairs could still be well clustered with noise below 10°, demonstrating the tolerance of the NEMO-TOC algorithm to alignment errors (Supplementary Figure S11). For the neuron samples we used in this work, the worst aligned ribosome conformation had an alignment error of 2.31°, which is well within the suitable range for NEMO-TOC (Supplementary Figure S11).

The topological organization of macromolecules is usually accompanied by conformational or compositional differences, as in the case of membrane-bound ribosomes (Supplementary Figure S7). Topological analysis of macromolecules can provide valuable contextual information about the organization of superstructures and their heterogeneity. The NEMO-TOC algorithm was further specifically optimized for more complicated cases like ribosomal organization, where a model with polarity must be considered. Clustering, tracking, and polishing were performed with limited supervision, which minimized the potential bias of the structural profiling process.

Because of the invisibility of mRNA, the full tracking of complete polyribosomes *in situ* is currently impossible. *In vitro* approaches, such as methods based on atomic force microscopy (AFM), allow the detection of naked mRNA chains; however, the purification process may disrupt the fragile interactions between ribosomes (46). Moreover, the purified super complex may collapse or become denatured during sample preparation (47). Therefore, *in vivo* detection methods are superior in this regard. Indeed, our present work is more in favor of the hypothesis that the mRNAs are mostly embedded inside an assembly of ribosomes, because for most of the ribosomes at least two neighboring ribosomes could be found within the distance of the ribosome's diameter (Supplementary Figure S1A). Overall, with the improvement of cryo-ET throughput and resolution, our approach has great potential for the structural profiling of polysome topology. In addition to neurons, we applied our algorithm to profile the ribosomal topology of metk overexpressing *E. coli* cells (Supplementary Figure S12), demonstrating the robustness of the method.

For eukaryotic neuron samples, our results clearly indicate that ribosomes adopt nonstochastic topologies *in situ*. The nonstochastic unit, considered here as the constitutional blocks formed by ribosomes from the same transformation clusters, may serve as a regulatory module as well. A similar idea has been proposed in previous work using various approaches (46). In our work, within each block, translation elongation tends to be synchronized, as indicated by the ribosome conformational analysis (Supplementary Figure S13). For the different types of blocks, although the individual ribosomal conformations indicate that they are all translationally active (Supplementary Figure S5C), a translation rate variance may exist, as shown by the mRNA trajectory (Figure 2K). The detailed molecular mechanisms of translation regulation on the level of ribosomal blocks could be studied by applying a similar approach for different growth conditions.

Polyribosomes were initially observed and described as long uniform superstructures (9). Although long uni-transformational polysomes exist, they are relatively rare. Polysomes are assembled from structured multi-ribosomal modules. In the cytosol, the uni-transformational packing of helical polysomes results in an overall straight shape. The integration of other modules, especially di-ribosomes, allows closed-loop translation initiation, where translation may become more efficient because of the high recycling rate of ribosomes (48). Other factors, such as the crowdedness of the cell, may contribute to the changing of cluster modules. It could also be possible that the regulatory elements of mRNAs are located in the regions between different modules.

It has been reported that there is a kinetic advantage for protein synthesis on the ER over synthesis in the cytosol, but the underlying mechanism has not been established (49). Our transformation clustering results show that although all the clusters were translationally active, the membrane-bound clusters had smoother mRNA trajectories (Figure 2K), which may enhance the efficiency of translation. These findings are consistent with a previous analysis of ER microsome-bound polyribosomes (15).

During our preparation of this manuscript, a work focusing on prokaryote Mycoplasma pneumonia was submitted to BioRxiv (50). Due to the small size of the organism, the sample preparation was more straightforward and the averaged ribosome maps achieved near-atomic resolution. Based on these results, a detailed analysis of *in situ* translation dynamics was possible. However, the sparse distribution of ribosomes makes it a unique case even compared with other prokaryotes such as *E. coli*. Our work here provides a robust analysis workflow and reports the first detailed structural profile of eukaryotic higher-order ribosomal topologies *in situ* despite limited map resolution. Currently, cryo-ET is the only method that preserves polysome topology. With further improvement in resolution and an enlarged view of interest (51), our algorithm will contribute to structural polysome profiling and elucidation of the regulatory role of ribosomal topology.

## DATA AVAILABILITY

NEMO-TOC source code, a detailed tutorial, and an example dataset for polysome tracking are available for download at https://github.com/GuoQLabPKU/polysomeTracking. Two primary neuron tomograms have been deposited in the Electron Microscopy Data Bank (EMDB) as EMD-33072 and EMD-33073. The corresponding ribosome averages have been deposited in the EMDB as EMD-33077 to EMD-33081. The membrane-bound ribosome (Cluster 3 and Cluster 6) average has been deposited in the EMDB as EMD-33018. The direct reconstructions of neighboring ribosomes of Cluster 1 to Cluster 3 have been deposited in the EMDB as EMD-33074 to EMD-33076.

## Supplementary Material

gkac547_Supplemental_Files
