# Supplementary material for: A transformation clustering algorithm and its application in polyribosomes structural profiling
Source: Nucleic Acids Res. 2022 Jul 11;50(16):9001–11. doi: 10.1093/nar/gkac547 (PMC9458451; doi:10.1093/nar/gkac547)
Supplement: gkac547_Supplemental_Files [file gkac547_supplemental_files.zip › manuscript_20220530_SI.pdf]

Supplemental Figure Legends

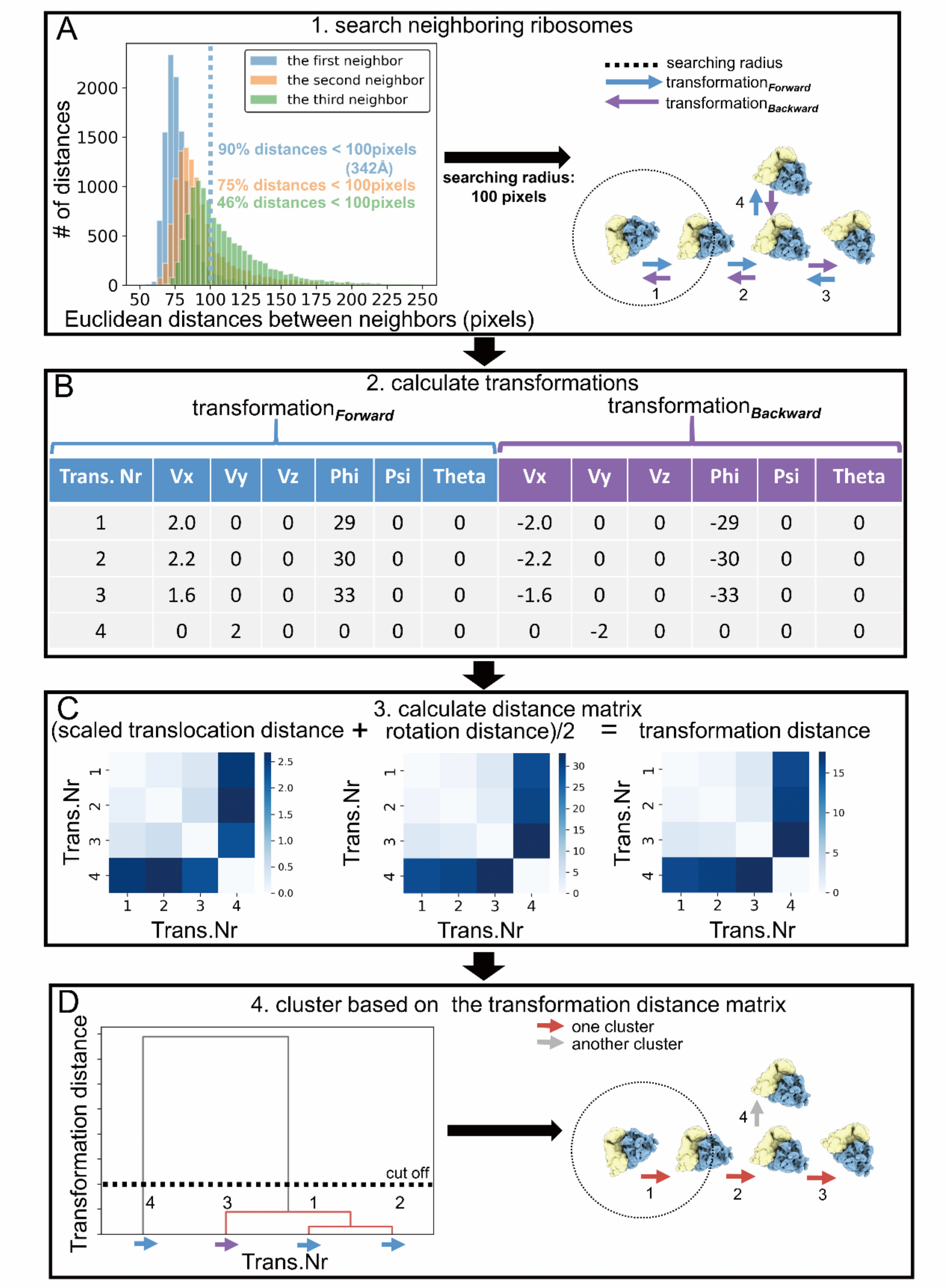

Figure S1. The workflow of NEMO-TOC

(A) The schematic diagram shows the definition of neighboring ribosome pairs. Firstly, as shown in the left panel, the distances between one ribosome and its neighbors were calculated. The nearest three distances are shown as blue, orange, and green bars. 100

pixels (342 Å) or roughly the diameter of one ribosome was used as the threshold for neighboring ribosome pair searching. The transformation of each pair was calculated including both forward (blue) and backward (purple) directions using both the positional and orientational information of each ribosome.

(B) Each transformation is composed of one translocation vector (Vx, Vy, Vz) and one rotation angle vector (Phi, Psi, Theta) with both forward (blue) and backward (purple) directions.

(C) The transformation distances between pairwise transformations were calculated (right matrix) as the combination of the scaled translocation distances (left matrix) and rotation distances (middle matrix). The distance matrixes are shown as heatmaps.

(D) The transformations are clustered based on the transformation distances from (C) using a hierarchical clustering method. For the case here, transformations 1 and 2 with the forward direction, and transformation 3 with the backward direction, are clustered into one cluster (colored in red). Transformation 4 has a transformation distance larger than those of the other transformations and is therefore moved to another cluster (colored in grey).

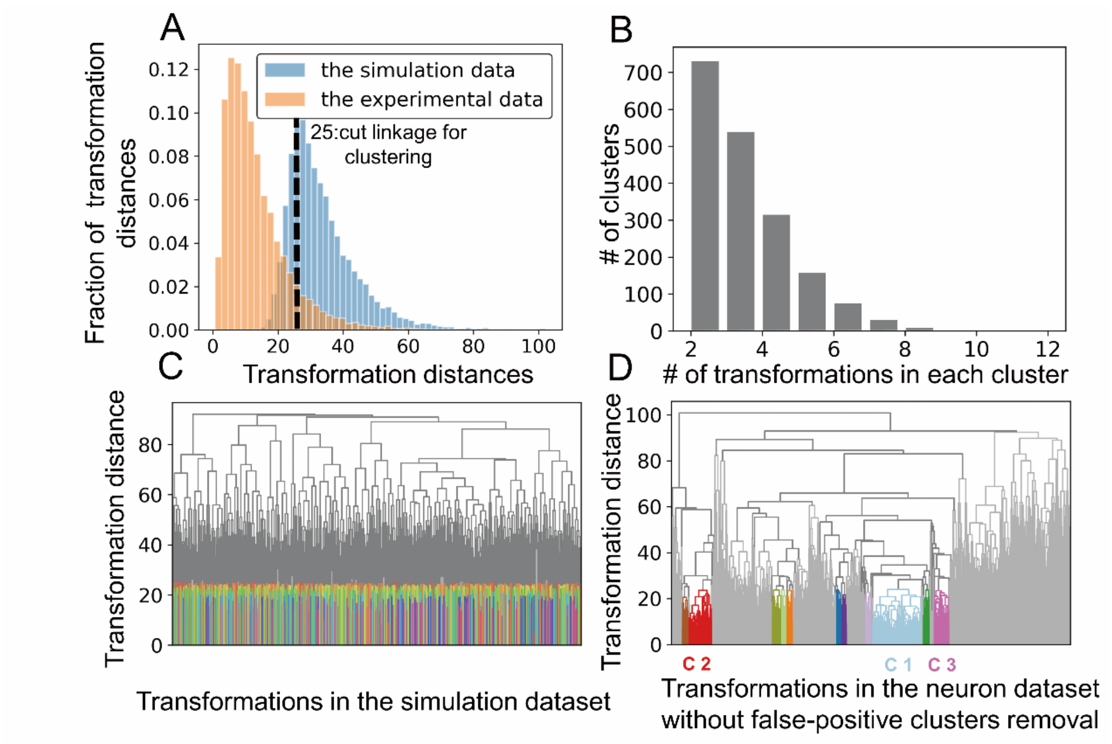

Figure S2. Metric to filter coincidentally grouped, non-meaningful clusters.

(A) The transformation distances from the simulated dataset with particles randomly rotated and positioned follow a positively skewed distribution, which tends to be larger than the distances from the experimental neuron dataset. The black dash line labels the value of 25, which was used for neuronal and *E. coli* dataset clustering, and only 10% of transformations have a smaller distance.

(B) The transformations were further clustered using the **NEMO-TOC** algorithm. The bar chart shows the distribution of transformation numbers after clustering. Only a few transformations were clustered together using the clustering parameter we adopted.

(C) The hierarchical clustering results of the neighboring ribosome transformations in the simulation data.

(D) The hierarchical clustering results of the neighboring ribosome transformations in the neuronal dataset without removal of non-meaningful clusters (colored in grey).

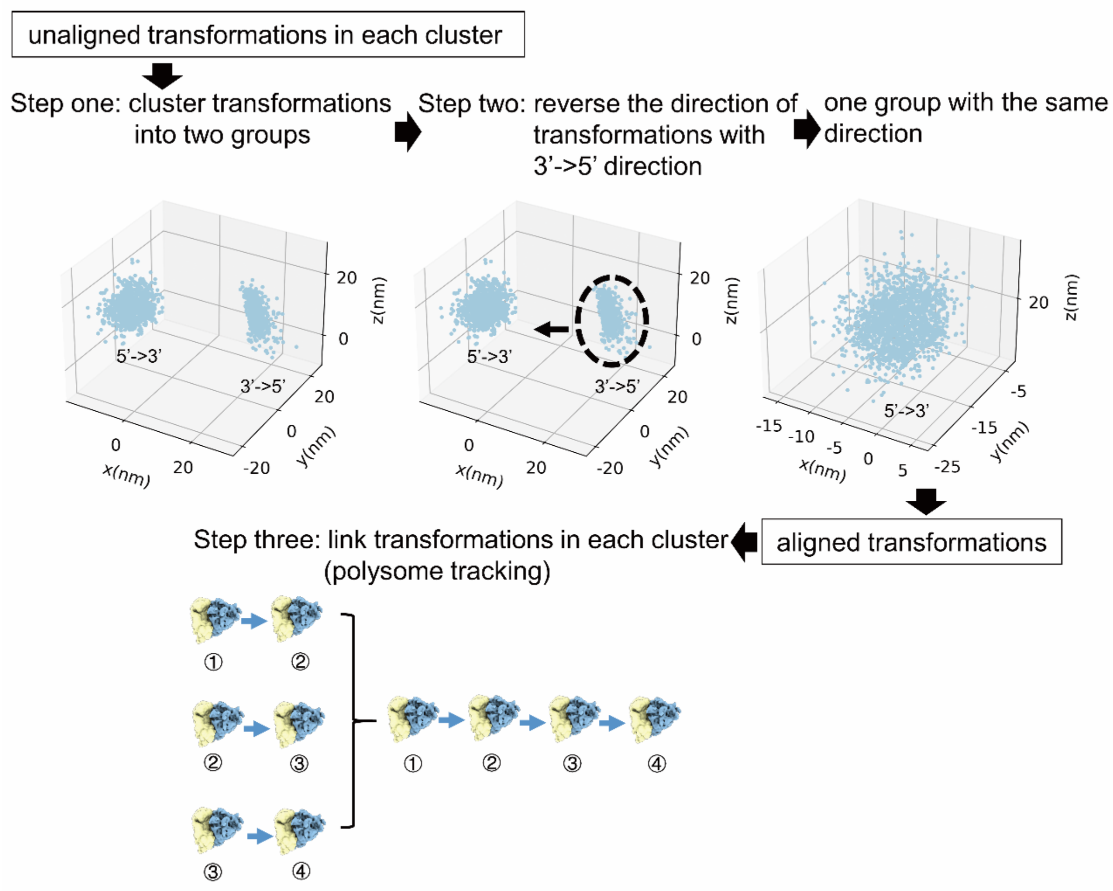

Figure S3. Homo-cluster polysome tracking.
Before polysome tracking, within each cluster, only the same consistent direction of each transformation should be kept. We define this step as direction alignment. To achieve this alignment, one direction was randomly selected for each transformation, resulting in two groups in the translocation vector space, representing two opposite directions (**Step one**). Then the direction representing the mRNA 3′->5′ direction was reversed, resulting in the same direction (mRNA 5′->3′) for each transformation (**Step two**). After direction alignment, all transformations have the same direction. Finally, connectable transformations that share the same ribosome are linked into a long chain, forming the polysome (**Step three**).

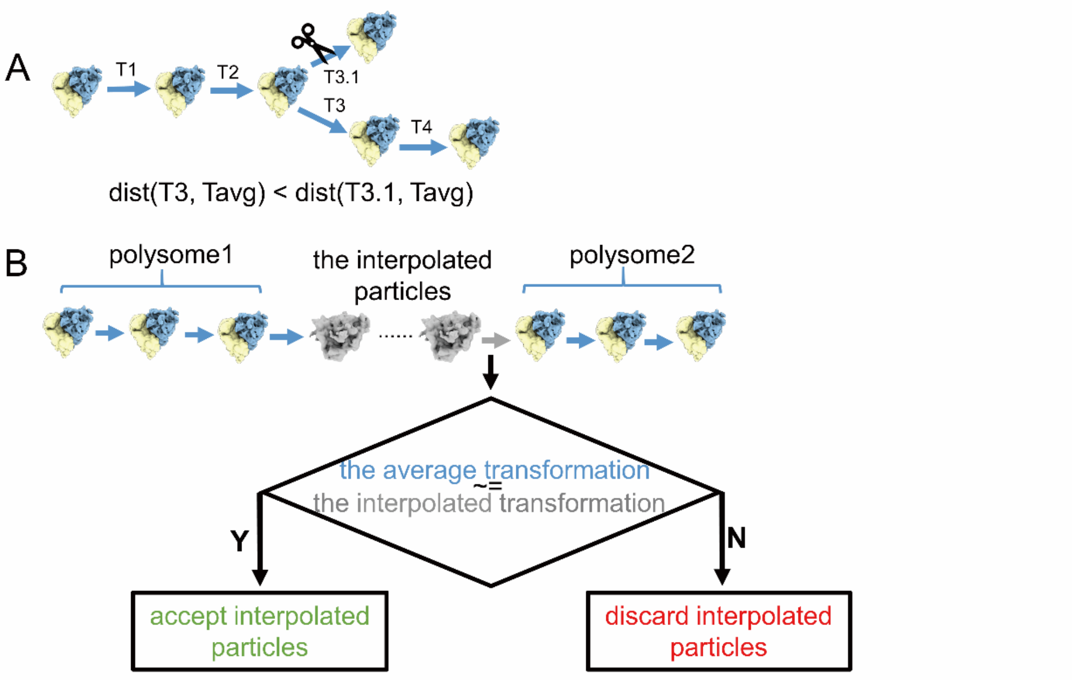

Figure S4. Design of polysome polishing

(A) The workflow of polysome branch removal. For polysomes with branches, the transformations that produced branches were searched, and only the transformation that had the smallest distance with the average transformation was kept.

(B) The workflow of polysome gap filling. To fill the gap caused by missing ribosomes, an interpolation-based approach was developed. For two polysomes formed by the same cluster, denoted polysome 1 and 2, up to five particles were first added one-by-one at the tail of polysome 1 using the average transformation. Then, the transformation between the last interpolated particle and the tip ribosome of polysome 2 was calculated. The interpolated particles were kept only if the transformation was similar to the average transformation of the cluster to which they belonged.

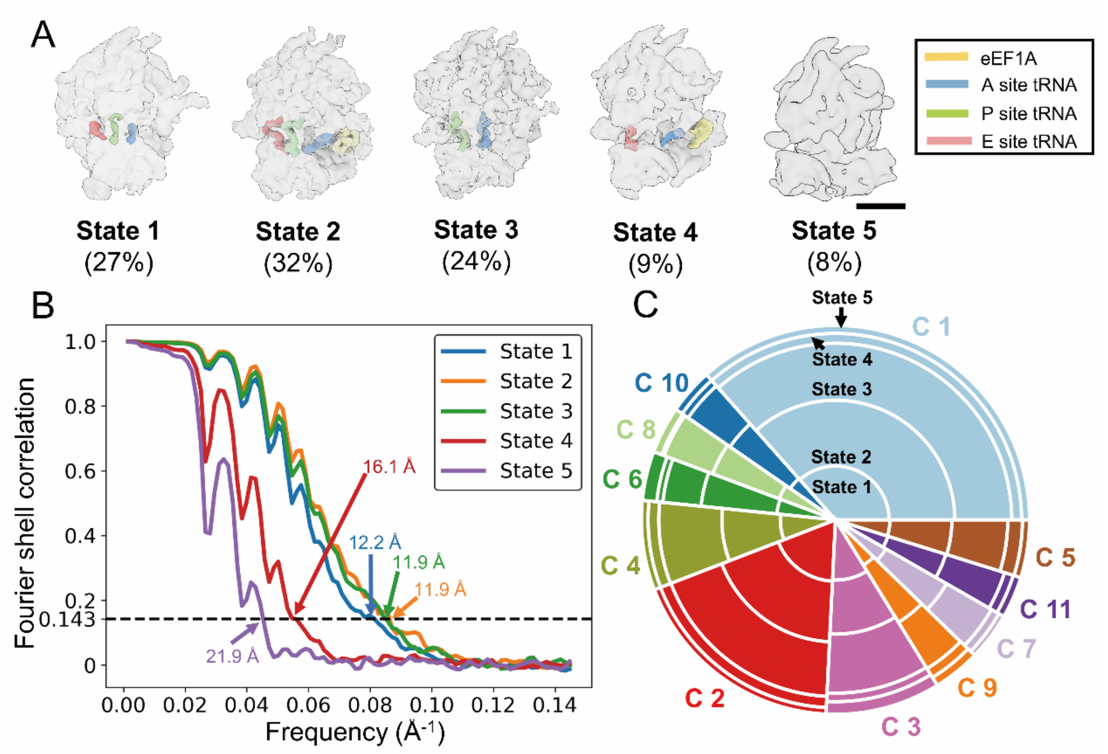

Figure S5. Conformational analysis of ribosomes from neuronal cells

(A) Five different ribosome conformations were detected in the neuron cells based on ratcheting status and tRNA site occupancy. The small and large subunits of the ribosome are represented as grey transparent surfaces, with densities representing eEF1A, A site tRNA, P site tRNA, and E site tRNA shown in yellow, blue, green, and red respectively. State 1 corresponds to the PRE-Classical-1/2 state. State 2 corresponds to the initial codon sampling state. State 3 corresponds to the PRE-Rotated-2 state. State 4 corresponds to the state with A and E site tRNA as well as eEF1A binding. State 5 is limited to lower resolution with no tRNA resolved. This limitation may be due to contamination by large subunit-only particles. Scale bar: 10 nm.

(B) Gold-standard Fourier shell correlation curves of the five conformation structures, yielding resolutions in the range between 12 Å to 22 Å using 0.143 as the cutoff value.

(C) The ribosome conformation distribution among the eleven transformation clusters. The arc length of each slice is proportional to the quantity of each transformation cluster, and the radius of the arc within each slice indicates the proportion of each conformational state.

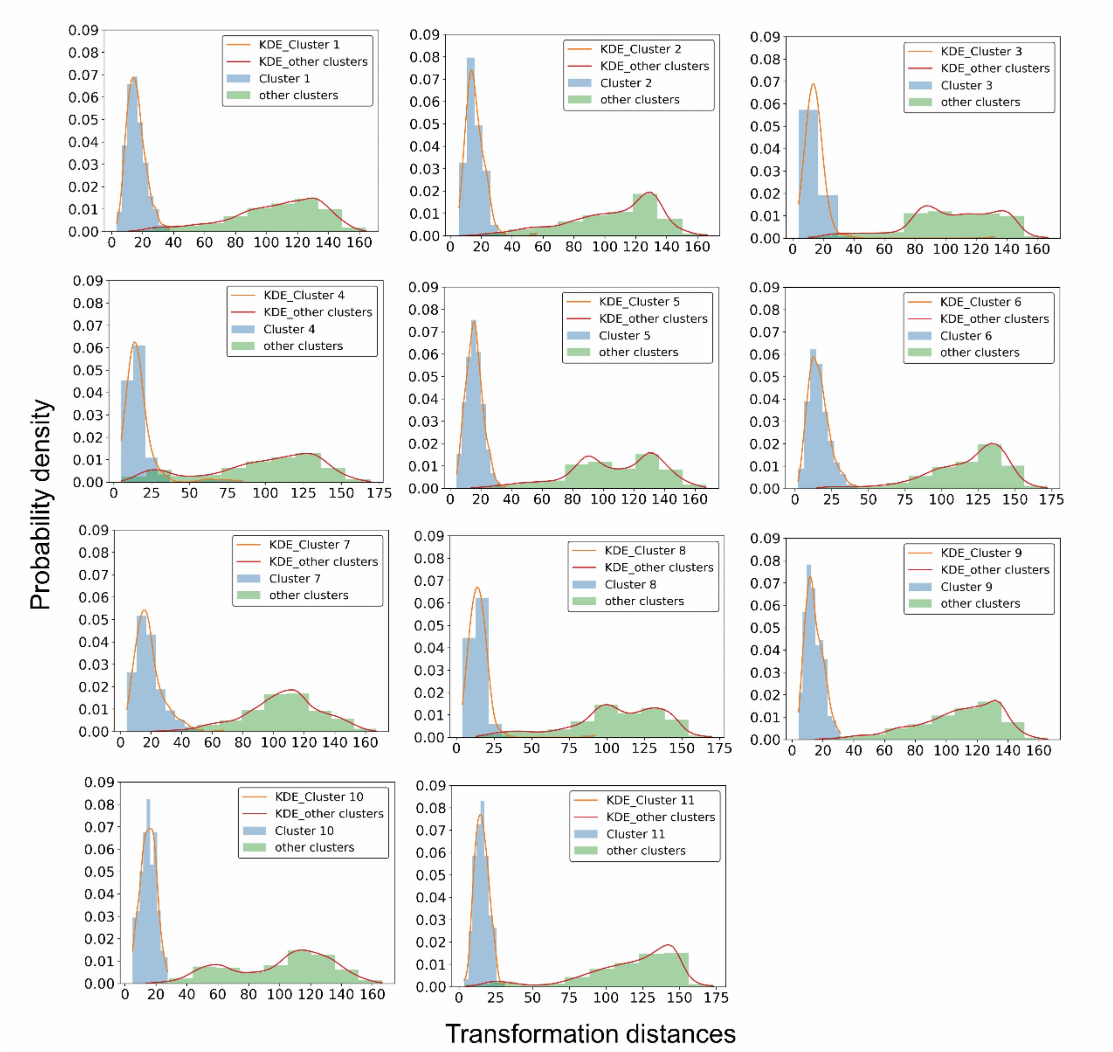

Figure S6. Comparison of transformation distances between transformations from each cluster and the remaining clusters

For each transformation cluster, the average transformation was calculated. The transformation distances were calculated between each transformation belonging to the same cluster and the average transformation, after which the probability density distribution (blue bars) was fitted (orange curves) to the results using the Gaussian kernel in the Python Scipy module. For comparison, the transformation distances between each transformation from the remaining clusters and the average transformation were also calculated, after which the probability density distribution (green bars) was fitted (red curves) to the results using the same Gaussian kernel in the Python Scipy module.

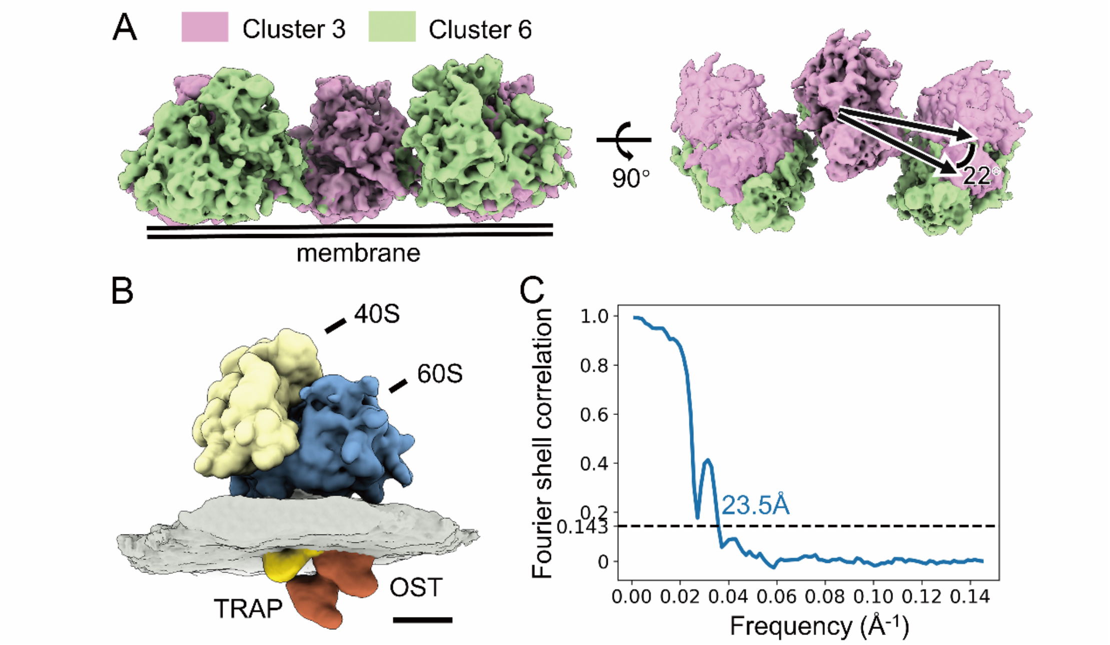

Figure S7. The structural features of transformation clusters of membrane-bound ribosomes

(A) Forward models of the two membrane-bound transformation clusters (Cluster 3 in pink and Cluster 6 in green) are shown in solid surface representations, aligned with the middle ribosome.

(B) Density map reconstructed with all 1,166 particles belonging to Clusters 3 and 6. The small subunit, large subunit, membrane, TRAP, and OST are colored in yellow, blue, grey, orange, and brown, respectively. Scale bar: 10 nm.

(C) Gold-standard Fourier shell correlation curve of the map shown in (B), yielding a resolution of 23.5 Å using 0.143 as the cutoff value.

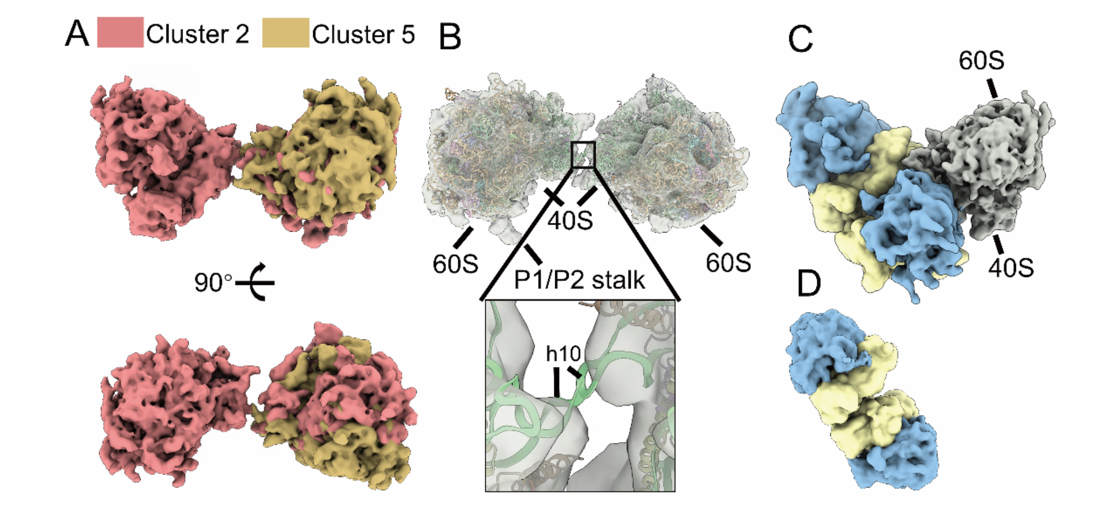

Figure S8. The structural features of transformation clusters of di-ribosomes

(A) Forward models of the two di-ribosome clusters (Cluster 2 in pink and Cluster 5 in brown) are shown in solid surface representations. The neighboring ribosome has a spatial shift in Cluster 2 compared with Cluster 5.

(B) The Cluster 2 forward model is shown as a transparent surface, with the atomic model (PDB:6QZP) superimposed on a ribbon representation. A close-up image of the indicated region shows that rRNA segment helix 10 (h10) is involved in the interaction.

(C) Surface representation of Cluster 2 forward model (grey) and a collision-stalled yeast di-ribosome (EMDB-4427, colored in yellow and blue for small and large subunits respectively), aligned with their upstream ribosome.

(D) Surface representation of a hibernating 100S ribosome from *E. coli* (EMDB-0139), with the small and large subunits colored in yellow and blue, respectively. The upstream ribosome was aligned to that of (C). All maps shown here were filtered to 15 Å for comparison.

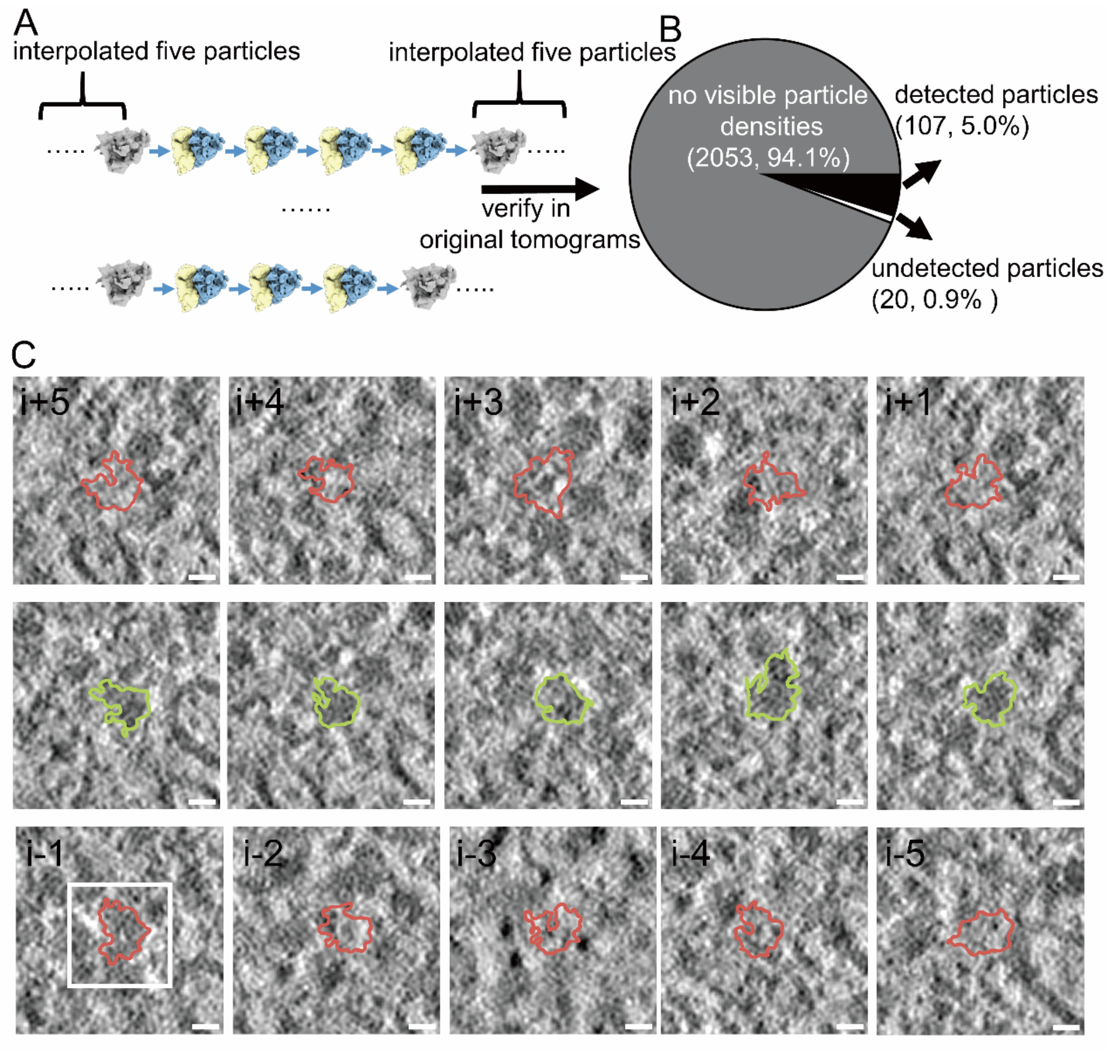

Figure S9. Assessment of missing particles

Analysis was performed to assess whether some particles from each polysome were missing because of false-negative detection during template matching. For polysomes formed by Cluster 1 that were longer than three ribosomes, five particles were interpolated at each side according to the calculated transformation (A). Then, we manually dissected the interpolated positions in the original tomograms. (B) As shown in the pie chart, of 2,180 interpolated particles, 94.2% are not visible in the tomograms, and 5.0% are classified as already detected but belong to different transformation clusters. Only 0.9% are indeed undetected particles. (C) An example shows the performance of the missing particle assessment. The central slices of ribosomes belonging to a polysome and the 10 interpolated ribosomes at both sides are shown here with the detected and predicted ribosome's position and shape represented as green and red strokes. The position that was confirmed to be a missing particle, which was not detected by template matching initially, is marked with a white rectangular box (labeled as i-1). Scale bar: 10 nm.

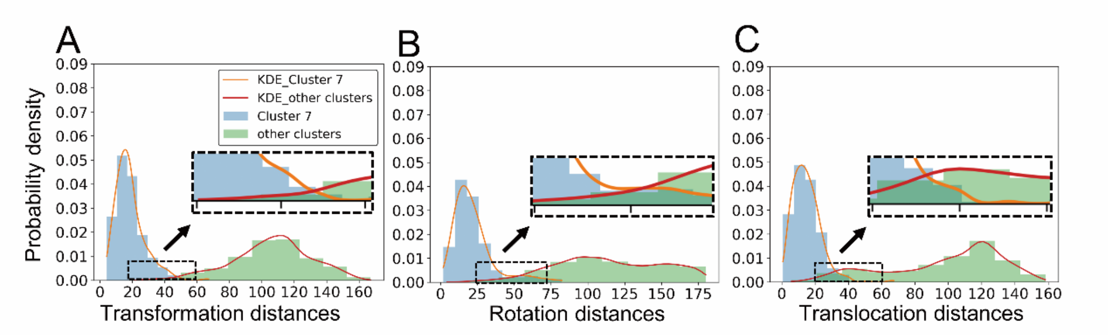

Figure S10. Comparision of clustering results using the transformation distance, rotation distance, and translocation distance

Using the method from Figure S6, the transformation distance distribution of the pairs from Cluster 7 were found to overlap less with the transformation distance distribution of pairs from other clusters (A) in comparison with the rotation distance distribution (B) and translocation distance distribution (C). The overlapped region is labeled by the dotted rectangles and shown magnified. The integral area of the overlapped region is 0.025, 0.100, and 0.085 for (A) (B), and (C) respectively.

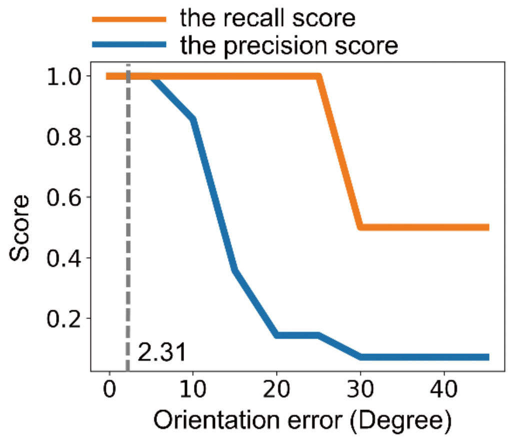

Figure S11 NEMO-TOC is tolerant to alignment error

To assess the effect of alignment error on NEMO-TOC, we generated a simulation dataset including a helical polyribosome formed by 15 ribosomes with 10 ribosomes randomly placed, which served as the background dataset. The final concentration of ribosomes is comparable with that of a typical biological sample. Additional random orientation errors were introduced to each ribosome to mimic alignment inaccuracy. After clustering, the recall score, which describes the fraction of neighboring polyribosome ribosomes pairs clustered into one cluster, and the precision score, which describes the fraction of clustered neighboring ribosomes pairs from the same polyribosome, were calculated under different inaccuracy alignments. Most ribosomes from the same polyribosome can be clustered together if the inaccurate orientation is less than 10°. The grey dashed line represents the angular alignment accuracy of neuronal ribosomes reported by RELION.

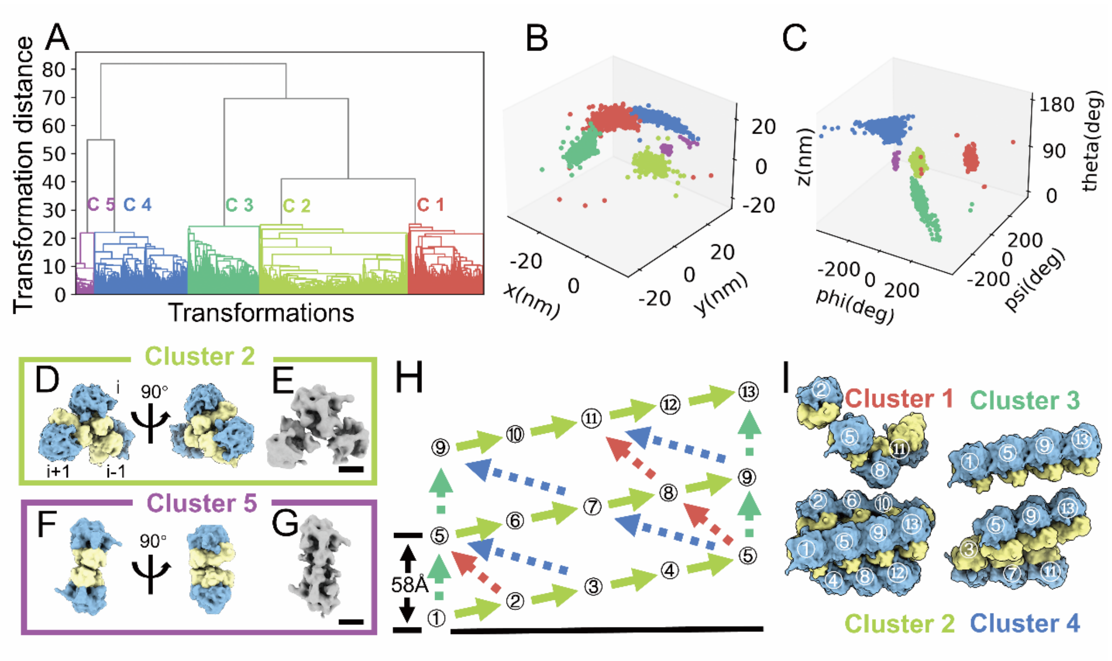

Figure S12. Transformation clustering of neighboring ribosomes in *E. coli*

(A) The hierarchical clustering results of the neighboring ribosome transformations. Five transformation clusters were clustered from the whole dataset. The five transformation clusters from (A) were well clustered in both the translocation vector space (B) and the rotation vector space (C). Forward models (D and F) and direct reconstructions (E and G) of two representative clusters are shown on a solid surface. Cluster 2 looks similar to the helix, forming a "top-top" conformation, while Cluster 5 forms a di-ribosome with packing identical with that of a hibernating 100S ribosome from *E. coli* (EMDB-0139). (H) Radical projection of the polysome helix formed by transformation Cluster 2, with a rise of 58 Å. Solid light green arrows denote the mRNA direction from 5′ to 3′. (I) Extended forward models of Clusters 1, 2, 3, and 4, reminiscent of the previously reported "spiral", "helical", "linear" and "zigzag" polysomes. As indicated by the dashed arrows in the radical projection (H), Clusters 1,3, and 4 are subsets of Cluster 2 that were formed by connecting neighboring ribosomes in different ways.

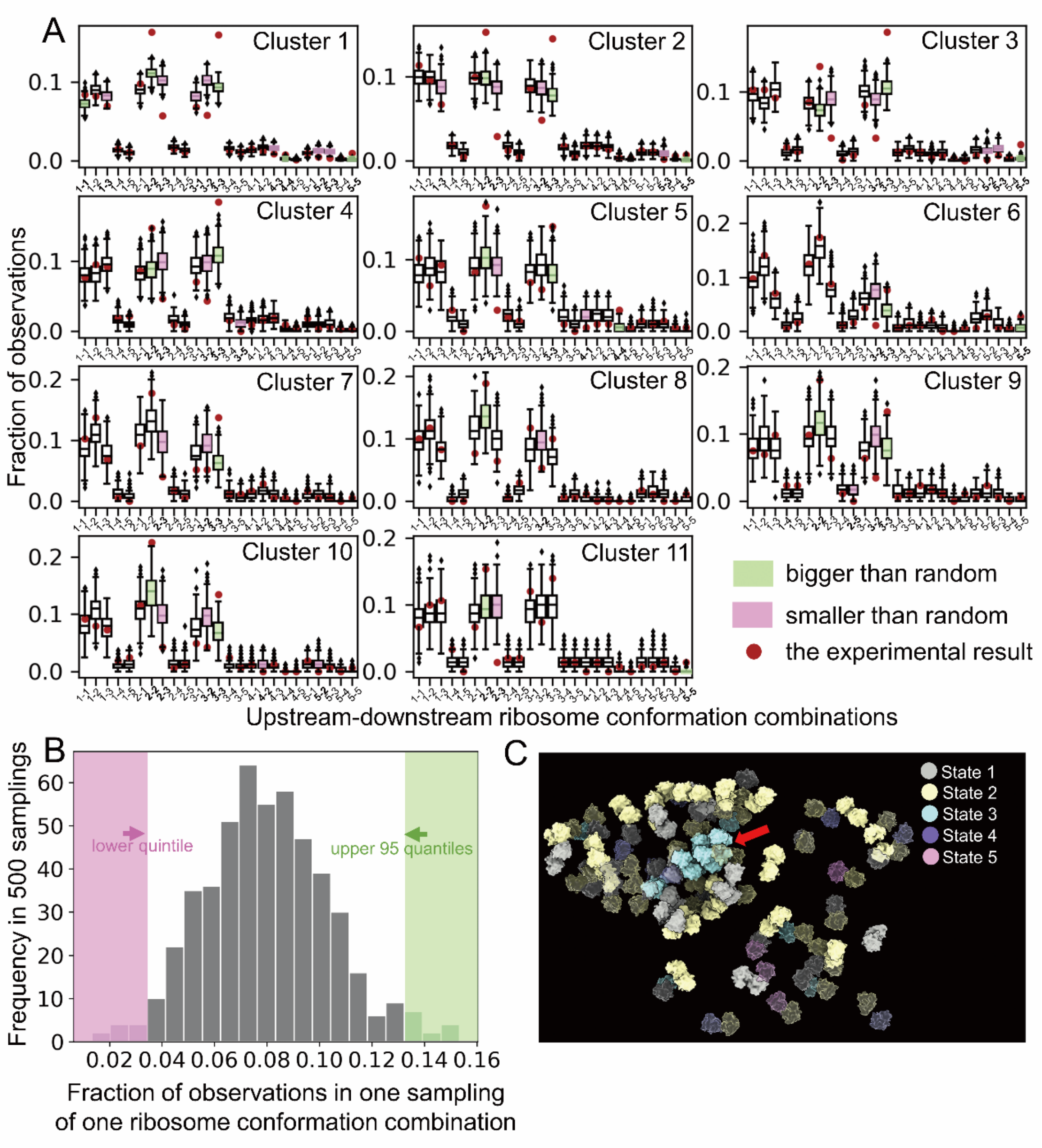

Figure S13. Clustered ribosomes tend to adopt the same conformation states

(A-B) We detected five different ribosome conformations. For each pair of ribosomes, there should be 25 (5x5) different combinations of upstream-downstream ribosomes. Here we explored whether these combinations are random within different clusters. In each cluster, we counted the occurrence fraction of each combination, which is shown as red dots (A). For comparison, we randomly sampled pairs of ribosomes from a ribosome pool with an identical conformation distribution and counted the occurrence fraction of each ribosome conformation combination. This process was repeated for 500 rounds, and the results are shown as box diagrams (A). For each fraction distribution, if the experimental occurrence fraction is smaller than the lower quintile (colored in pink), the experimental occurrence fraction is labeled as significantly smaller than random. If the experimental occurrence fraction is larger than the upper 95 quantiles (colored in green), the experimental occurrence fraction is labeled as significantly larger than random (B). For visualization, only the conformation state combinations that have experimental occurrence fractions

significantly different from the simulation results are labeled in bold in the x-axis (A). For all clusters except Clusters 6 and 7, if the upstream ribosome adopts conformation State 2, its downstream ribosome also tends to have the identical conformation. For all clusters except Clusters 8 and 11, if the upstream ribosome adopts conformation State 3, its downstream ribosome also tends to be identical. (C) One representative tomogram with all ribosomes reintroduced and colored based on their conformations. If the upstream ribosome and its downstream ribosome have the same conformation state, the ribosomes are colored without any transparency. The red arrow denotes the longest cytoplasmic polysome in which the majority of ribosomes are synchronized in conformation State 3.

Other Supplemental Items

Table S1. Cluster summary

stdTransVect: standard deviation of the translocation vector compared with the average
stdTransAng: standard deviation of the rotation vector compared with the average
meanTransVectX: average translocation vector X
meanTransVectY: average translocation vector Y
meanTransVectZ: average translocation vector Z
meanTransAngPhi: average rotation angle phi
meanTransAngPsi: average rotation angle psi
meanTransAngTheta: average rotation angle theta

clusterNr	# of particles	stdTransVect	stdTransAng	meanTransVectX	meanTransVectY	meanTransVectZ	meanTransAngPhi	meanTransAngPsi	meanTransAngTheta
**1**	3124	13.98	21.77	-10.37	-40.89	54.94	-136.13	174.33	72
**2**	1569	14.5	23.02	11.07	84.11	31.83	112.54	56.96	97.55
**3**	817	19.08	26.06	22.81	72.6	-6.28	23.36	-20.57	25.41
**4**	637	15.51	20.97	-33.13	4.07	70.91	-60.54	29.28	73.7
**5**	410	14.46	20.52	13.48	85.77	22.46	123.92	47.84	64.87
**6**	349	28.46	20.2	-16.97	-44.95	62.49	-163.02	164.8	69.89
**7**	341	14.9	18.44	54.04	37.32	34.15	-28.04	-32.02	92.89
**8**	336	17.52	21.04	73.31	-23.21	-1.92	142.15	-139.65	73.3
**9**	336	14.99	21.61	81.08	-12.67	3.56	115.99	-117.82	67.89
**10**	324	19.26	18.79	55.24	44.03	28.62	45.98	-47.11	88.1
**11**	297	15.72	18.83	-39.16	-0.18	65.6	-143.88	112.79	67.83

Table S2. Summary of the number of overlapping particles among different clusters

pairCluster: one pair of clusters

riboNr_C1: number of ribosomes in the first cluster

riboNr_C2: number of ribosomes in the second cluster

overlapRiboNr: number of overlapping ribosomes between two clusters

pairCluster	riboNr_C1	riboNr_C2	overlapRiboNr
**C 1: C 2**	3124	1569	322
**C 1: C 3**	3124	817	62
**C 1: C 4**	3124	637	161
**C 1: C 5**	3124	410	67
**C 1: C 6**	3124	349	21
**C 1: C 7**	3124	341	51
**C 1: C 8**	3124	336	87
**C 1: C 9**	3124	336	36
**C 1: C 10**	3124	324	56
**C 1: C 11**	3124	297	73
**C 2: C 3**	1569	817	4
**C 2: C 4**	637	1569	166
**C 2: C 5**	1569	410	0
**C 2: C 6**	349	1569	8
**C 2: C 7**	1569	341	21
**C 2: C 8**	336	1569	114
**C 2: C 9**	1569	336	141
**C 2: C 10**	324	1569	34
**C 2: C 11**	1569	297	21
**C 3: C 4**	637	817	0
**C 3: C 5**	817	410	1
**C 3: C 6**	349	817	99
**C 3: C 7**	817	341	2
**C 3: C 8**	336	817	1
**C 3: C 9**	817	336	0
**C 3: C 10**	324	817	3
**C 3: C 11**	817	297	1
**C 4: C 5**	637	410	16

**C 4: C 6**	349	637	3
**C 4: C 7**	637	341	12
**C 4: C 8**	336	637	9
**C 4: C 9**	637	336	19
**C 4: C 10**	324	637	23
**C 4: C 11**	637	297	11
**C 5: C 6**	349	410	3
**C 5: C 7**	341	410	9
**C 5: C 8**	336	410	15
**C 5: C 9**	336	410	12
**C 5: C 10**	324	410	1
**C 5: C 11**	297	410	3
**C 6: C 7**	349	341	0
**C 6: C 8**	336	349	2
**C 6: C 9**	349	336	2
**C 6: C 10**	324	349	1
**C 6: C 11**	349	297	3
**C 7: C 8**	336	341	3
**C 7: C 9**	336	341	8
**C 7: C 10**	324	341	6
**C 7: C 11**	341	297	7
**C 8: C 9**	336	336	10
**C 8: C 10**	324	336	5
**C 8: C 11**	336	297	1
**C 9: C 10**	324	336	7
**C 9: C 11**	336	297	8
**C 10: C 11**	324	297	3

Movie S1. Forward models of the ribosome transformation clusters.
